# Clinical Validation of the Defining Characteristics of the Nursing Diagnosis ‘Activity Intolerance’ in Patients With Acute Coronary Syndrome

**DOI:** 10.1002/nop2.70050

**Published:** 2024-12-05

**Authors:** Diana Isabel Cáceres Rivera, Luz Mileyde Jaimes Rojas, Luisa Yaneth Cristancho Zambrano, Jennifer Vanesa Acosta Barón, Diana Ivonne Cañon Gómez, Lyda Z. Rojas

**Affiliations:** ^1^ Research Group for the Strengthening of Health and Wellness (GIFOSABI) Universidad Cooperativa de Colombia Bucaramanga Colombia; ^2^ Research Group and Development of Nursing Knowledge (GIDCEN‐FCV) Research Center, Fundación Cardiovascular de Colombia Floridablanca Colombia; ^3^ Acute Myocardial Infarction Center of Excellence Fundación Cardiovascular de Colombia Floridablanca Colombia

**Keywords:** Coronary artery disease, nursing diagnosis, validation study

## Abstract

**Aim:**

To validate the defining characteristics (CDs) of the nursing diagnosis ‘Activity intolerance [00092]’ in patients with acute coronary syndrome (ACS).

**Design:**

Cross‐sectional study. Patients over 18 years of age with a confirmed medical diagnosis of hospitalised ACS were included and those who were haemodynamically unstable, with alterations in the mental sphere or with communication limitations were excluded. The CDs were previously operationalised for standardised measurement and independently assessed by nurses.

**Results:**

A total of 111 patients with ACS were studied. The CDs with prevalence > 50% were: generalised weakness (80.56 vs. 78.70%), discomfort on exertion (72.07% vs. 75.68%) and ECG changes (71.17% vs. 68.47%). Inter‐rater agreement for determining the CDs ranged from 0.69 to 1.00.

**Conclusions:**

This study established the clinical validation of the CDs of the nursing diagnosis ‘Activity Intolerance [00092]’ in patients with ACS identifying three major CDs: EKG changes, generalised weakness and exertional discomfort.

**Patient or Public Contribution:**

No patient or public contribution the research was developed in its entirety by the authors.

**Implications for the Profession and/or Patient Care:**

- The clinical validation of the defining characteristics of the activity intolerance diagnosis was conducted. This validation was based on operationalising each characteristic using commonly employed nursing scales and instruments. This process contributes significantly to the establishment of evidence‐based nursing practices.
- The customisation of nursing diagnoses gains further ground through the validation studies of NANDA‐I diagnoses. This process solidifies standardised nursing language as a valuable strategy in nursing care, providing substantial support for practical decision‐making.
- Among the evaluators, EKG changes, generalised weakness and discomfort on exertion emerged as the most frequently encountered defining characteristics with substantial agreement. This discovery offers crucial insights for devising individualised and collective care plans within coronary units.

## 1 Introduction

Cardiovascular diseases (CVD) stand as the foremost global cause of mortality, claiming approximately 17.9 million lives annually (World Health Organization 2022). Among these fatalities, four out of every five are attributed to heart attacks and strokes. In Colombia, acute coronary syndrome (ACS) accounted for the death of 16,000 men out of a total of 9000 deaths in 2011 (Avezum et al. 2009). Similarly, in 2020 and 2021, following the aftermath of COVID‐19, cardiovascular diseases maintained their position as the second principal cause of death. Within this category, AMI constituted 60% of these deaths, akin to the figures for COVID‐19 (Ministry of Health and Social Protection 2022).

Activity intolerance has been described among other physiological findings of ACS (Paiva and de Oliveira Lopes 2005). This human response is defined by the North American Nursing Association (NANDA) as ‘the lack of sufficient physiological or psychological energy to tolerate or complete required or desired daily activities’ and belongs to domain 4 activity/rest, Class 4 cardiovascular/pulmonary responses, need: moving and pattern: activity exercise (NANDA International n.d.).

The pathophysiology of activity intolerance in ACS stems from an imbalance between the myocardium's energy supply and demand. As a result, ischemia leads to a reduction in the contractile capacity of muscle fibers. This reduction can initiate a cascade of signs and symptoms, as outlined in the defining characteristics of this nursing diagnosis. These include alterations in heart rate, electrocardiographic changes, fatigue and more (NANDA International n.d.; Puntunet 2008; Moreu‐Burgos 2007).

This diagnosis has predominantly been validated in patients with heart failure, (de Souza 2014; Souza 2015; Pereira et al. 2016) other cardiovascular diseases, and patients with refractory angina (Mol and Baker 1991; Zampieron, Aldo, and Corso 2011; Cardoso et al. 2019). However, concerning our context, only Rodrigues et al. clinically validated the defining characteristics (DC) of the nursing diagnosis ‘Intolerance to activity’ in patients with ischaemic heart disease (Rodrigues et al. 2011, 2012). No validation studies of this diagnosis in ACS were found.

The importance of research on nursing diagnoses in each country lies in the need to unify criteria in the same context, to contribute to the development of consolidated and verifiable registries, and finally to maintain a continuous change that reflects the dynamics of nursing care and clinical practice (Lunney 2008). Therefore, the aim of this study was to clinically validate the defining characteristics of the nursing diagnosis ‘Intolerance to activity [00092]’ in patients with ACS, hospitalised in a fourth‐level care centre specialised in cardiovascular health.

## 2 Methods

### 2.1 Study Design

This cross‐sectional study adheres to the Strengthening the Reporting of Observational Studies in Epidemiology (STROBE) statement (Elm et al. 2007).

### 2.2 Setting

This study was conducted at the Intermediate Intensive Care Units and Medical hospitalization Units in Fundación Cardiovascular de Colombia (FCV) in Floridablanca, Santander, Colombia from October 2019 to December 2021. These dates acompassed periods of suspension due to the health emergency stemming from the COVID‐19 pandemic.

### 2.3 Participants

The study included adult patients admitted to the hospital with a confirmed medical diagnosis of ACS.

### 2.4 Inclusion Criteria

Patients aged 18 years and above with a confirmed medical diagnosis of ACS were included.

### 2.5 Exclusion Criteria

Patients who were hemodynamically unstable, experiencing alterations in mental state, or possessing communication limitations that hindered their participation in the interview were excluded.

### 2.6 Sample Size

Initially, a sample size was calculated based on the following parameters: an average daily admission of around two to three ACS cases (totalling 450 events in the last 6 months) expecting a prevalence of DCs of the nursing diagnosis ‘activity intolerance’ of 50%, a confidence level of 95% and an alpha of 5%. This calculation resulted in a sample size of 208 participants. However, due to the same COVID‐19 emergency, a recalculation was performed based on preliminary findings. Consequently, the expected prevalence of DCs was adjusted to 10% while retaining and the same alpha and confidence were maintained for a total of 106 participants needed. This adjustment yielded a required sample size of 106 participants. The sample was selected through a nonprobabilistic approach. Participants were consecutively included as they were identified during daily medical rounds by the research staff's cardiologist, who verified adherence to the inclusion and exclusion criteria.

### 2.7 Data Collection

Two pairs of Registered Nurses were trained two professionals were part of the research team, while the other two were affiliated with the institution where sample collection took place, albeit from a different ICU than the one where data was gathered. To ensure data reliability, the following criteria were considered: a minimum of 5 years' experience in the ICU, postgraduate education (specialization, master's or doctorate) and research experience.

Each couple was responsible for managing informed consent, recording sociodemographic information, personal history and clinical characteristics pertaining to the patient's ACS. Data collection was carried out using the Commcare application (ref comment), with a minimum interval of 2 h between both measurements.

### 2.8 Outcome

The training nurses assessed the defining characteristics of the nursing diagnosis ‘Activity Intolerance’ using a format devised by the researchers. This format, in addition to encompassing clinical and sociodemographic details, evaluated the presence or absence of these defining characteristics. Operationalization of these characteristics was executed through specific measurement scales or instruments, chosen following an extensive literature review. This review included articles on the validation of various nursing diagnoses and underwent scrutiny by professionals boasting extensive nursing and cardiology experience (Cáceres et al. 2022).

### 2.9 Correlate

A clinical validation, following the Fehring proposal, was undertaken. This model seeks to gather evidence for the existence of a particular diagnosis directly from the clinical setting. The model operates through clinical observation, wherein two expert clinicians conduct the observations and evaluations. The modified CDV model can adopt this approach or involve acquiring clinical information directly from the patient subject. The choice of approach depends on the diagnosis's nature being tested. For diagnoses more aligned with performance or physiology, such as in our case, a direct observation method is fitting. Before implementing this model, it is essential to meticulously describe each defining characteristic of the diagnosis under examination. If possible, operational definitions should be formulated for each defining characteristic. Ultimately, the weighted inter‐rater reliability coefficients for each defining characteristic are computed using a specific formula. The weighted interrater reliability ratio for each defining characteristic is determined using a dedicated formula (Fehring 1987; Grant, Kinney, and Guzzetta 1990; Whitley 1997).

### 2.10 Statistical Analysis

A descriptive analysis was performed, the variables in qualitative measurement scale were described as absolute and relative frequencies; the continuous variables were described with measures of central tendency and dispersion according to their characteristics; the continuous variables with normal distribution were summarised with the mean and standard deviation and those that did not present a normal distribution were described with the median and first and third quartiles.

Concerning the defining characteristics of the nursing diagnosis, their prevalence was estimated, and the level of agreement between the two evaluating nurses was assessed utilizing the Kappa index. For major characteristics, those with a prevalence equal to or surpassing 50%, and for minor characteristics, those below 50% were considered. Furthermore, the degree of agreement was categorised as very good within the range of 0.8–1.0, good within 0.6–0.8, moderate within 0.4–0.6, poor within 0.2–0.4, and negligible within 0.0–0.2 (Abraira 2001).

### 2.11 Ethical Considerations

This study was approved by the Research Ethics Committee of the Fundación Cardiovascular de Colombia, in accordance with national and international guidelines for research in humans. All participants gave their written informed consent after being fully informed about the objectives and development of the project.

## 3 Results

A total of 111 patients were included. The participants had an average age of 65 years (SD ± 10). Of these, 67.57% (*n* = 75) were male, 72.07% (*n* = 80) hailed from rural areas, and 74.77% (*n* = 83) belonged to the low socioeconomic stratum. Marital status distribution was as follows: 36.04% (*n* = 40) were married, followed by common‐law, single, widowed and divorced. Educational background revealed that 63.07% (*n* = 70) had no schooling, incomplete or complete primary education. Regarding social security, 63.06% (*n* = 70) were under a subsidised system, and 82.88% (*n* = 92) had been referred (Table 1).

**TABLE 1:** Sociodemographic characteristics of hospitalised patients with acute myocardial infarction (*n* = 111).

Features	*n* (%)
Sex	
Male	75 (67.57)
Woman	36 (32.43)
Age (years)^a^	65 ± 10.2
Area of origin	
Urbana	80 (72.07)
Rural	31 (27.93)
Socioeconomic stratum	
Low	83 (74.77)
Medium	28 (25.23)
Marital status	
Single	19 (17.12)
Divorced	7 (6.31)
Married	40 (36.04)
Widower	18 (16.22)
Free union	27 (24.32)
Schooling	
None	7 (6.31)
Incomplete elementary school	41 (36.94)
Completed elementary school	22 (19.82)
Incomplete baccalaureate	15 (13.51)
Completed baccalaureate	10 (9.01)
Complete technology	4 (3.60)
University incomplete/complete	12 (10.81)
Occupation	
Independent	52 (46.85)
Employee	13 (11.71)
Unemployed	22 (19.82)
Retired	18 (16.22)
Another	6 (5.41)
Social security	
Contributory	36 (32.43)
Subsidised	70 (63.06)
Prepaid	1 (0.90)
Special regime	4 (3.60)
Forwarded to	
Yes	92 (82.88)
No	19 (17.12)

^a^ Mean and standard deviation.

In relation to clinical characteristics, the most prevalent personal history was sedentary lifestyle (67.57%) followed by hypertension (59.46%), dyslipidaemia (58.56%), angina pectoris (43.24%) and diabetes mellitus (32.43%). A total of 19 patients (17.12%) were classified under NYHA functional Class III–IV; in the electrocardiogram 28.83% (*n* = 32). In the electrocardiogram, 32 participants (28.83%) exhibited pathological Q waves, while 56 (50.45%) displayed segment changes. Additionally, 50% of the participants had two or more obstructed vessels, and their median ejection fraction was 49% (Q1 = 35; Q3 = 55). Regarding pharmacological treatment, 105 individuals (94.59%) were on beta‐blockers, 95 (85.59%) were taking statins, 94 (84.68%) were using antiplatelet agents, and 88 (79.28%) were prescribed ACEI/ARA II, among other treatments (Table 2).

**TABLE 2:** Clinical characteristics of hospitalised patients with acute myocardial infarction (*n* = 111).

Features	*n* (%)
Personal background	
Sedentary lifestyle	75 (67.57)
Hypertension	66 (59.46)
Dyslipidaemia	65 (58.56)
Angina pectoris	48 (43.24)
Diabetes mellitus	36 (32.43)
Obesity	29 (26.13)
Old IAM	26 (23.42)
Thyroid disease	10 (9.01)
Acute/chronic renal failure	9 (8.10)
CVD/CCA	8 (7.21)
Peripheral vascular disease	8 (7.21)
Arrhythmia	8 (7.21)
Heart failure	7 (6.31)
Smoking rate ex‐smoker (*n* = 52)	14.2 ± 13.9 package/year
Smoking rate current smokers (48)	10.2 ± 17.4 package/year
Alcohol consumption	42 (37.84)
NYHA functional class	
I–II	92 (82.88)
III–IV	19 (17.12)
EKG changes	
Pathological Q waves	32 (28.83)
ST segment changes	56 (50.45)
Number of vessels affected (obstruction)	2 (Q1 = 1; Q3 = 3)
0	15 (13.51)
1	24 (21.62)
2	17 (15.32)
3	47 (42.34)
Multivessel	8 (7.21)
LVEF (%)	49 (Q1 = 35; Q3 = 55)
Pharmacological treatment	
Beta blocker	105 (94.59)
Statins	95 (85.59)
Platelet antiaggregant	94 (84.68)
IECA/ARA II	88 (79.28)
Anticoagulants	72 (64.86)
Aldosterone	28 (25.23)
Diuretics	22 (19.82)
Antiarrhythmics	6 (5.41)
Analgesics	4 (3.60)
Vasodilators	2 (1.80)
Inotropics	2 (1.80)

### 3.1 Clinical Validity of the Defining Characteristics of the Nursing Diagnosis Activity Intolerance [00092]

Concerning the clinical validity of the nursing diagnosis ‘Activity Intolerance’ [00092], three defining characteristics were identified as major, each with prevalence exceeding 50%: Generalised weakness (80.56% vs. 78.70%), exertional discomfort (72.07% vs. 75.68%), and ECG changes (71.17% vs. 68.47%). Interjudge agreement was nearly perfect for the defining characteristics exertional dyspnoea (*k* = 1.000) and fatigue (*k* = 1.000), and substantial for heart rate (*k* = 0.729) and abnormal systolic blood pressure (*k* = 0.718). Agreement was moderate for ECG changes (*k* = 0.594) and abnormal diastolic pressure (*k* = 0.592), and finally, agreement was poor/mild for exertional discomfort (*k* = 0.207; Table 3).

**TABLE 3:** Prevalence and concordance of the clinical validation of the defining characteristics of the nursing diagnosis of activity intolerance [00092] (*n*=111).

Defining characteristics	Judge 1 *n* (%)	Judge 2 *n* (%)	Agreement (%)	Expected agreement (%)	Kappa (*k*)
ECG changes (arrhythmias, conduction abnormality, ischemia)	79 (71.17)	76 (68.47)	82.88	57.82	0.594
Generalised weakness	87 (80.56)	85 (78.70)	96.23	67.09	0.885
Stress discomfort	80 (72.07)	84 (75.68)	69.37	61.33	0.207
Dyspnoea on exertion	44 (39.64)	44 (39.64)		52.15	1.000
Abnormal heart rate in response to activity	23 (21.30)	18 (16.51)	91.59	68.91	0.729
Abnormal arterial diastolic pressure in response to activity	13 (12.04)	12 (11.11)	91.51	79.19	0.592
Abnormal arterial systolic pressure in response to activity	11 (10.19)	13 (12.04)	94.34	79.90	0.718
Fatigue	8 (7.21)	8 (7.21)		86.62	1.000

## 4 Discussion

In this cross‐sectional study, we conducted a clinical validation of the defining characteristics of the nursing diagnosis ‘Activity Intolerance [00092]’ among hospitalised patients in a specialised fourth‐level care center for cardiovascular health. Our findings identified three major defining characteristics: (1) EKG changes, (2) generalised weakness and (3) discomfort on exertion. Additionally, we found other characteristics, such as ECG changes and abnormal arterial diastolic pressure in response to activity, classified as minor. All identified characteristics demonstrated satisfactory agreement, validating their incorporation into nursing care plans.

Activity intolerance has received limited assessment, despite its frequent occurrence in patients with cardiovascular disease. Studies in Brazil and Colombia have established both the risk and prevalence of this nursing diagnosis in patients with acute coronary syndrome (Padilla‐Garcia et al. 2017; Dias Emidio et al. 2021). However, the deeper validation of defining characteristics, as a crucial initial step for accurate diagnosis, remains largely unexplored.

Similarly, in 2011, Rodrigues et al. clinically validated the defining characteristics of Activity Intolerance in 22 patients with ischemic heart disease and refractory angina. Their findings revealed EKG changes indicating ischemia, verbal reports of fatigue and abnormal heart rates in response to physical activity as major defining characteristics. The interobserver reliability indices for most of these characteristics ranged between 0.5 and 0.79. Differences in their results could be attributed to patient recruitment settings (outpatient vs. clinic), with similarities in EKG findings despite setting variations, particularly in the persistence of Q waves beyond the acute phase (Odenas Alesina et al. 2021). Furthermore, limitations in sample size and employed methodology (expert‐based Fehring approach vs. clinic‐based operationalisation of defining characteristics) may have influenced accuracy and objectivity in determining defining characteristics (Rodrigues et al. 2011).

Regarding the most prevalent feature, ECG changes, it is noteworthy that noninvasive electrocardiography remains the most cost‐effective option for continuous monitoring of coronary patients, both pre‐ and in‐hospital (Siontis et al. 2021; Meek and Morris 2002). This tool yields prognostic information crucial for timely determination of reperfusion status in ACS or new occlusions (Strong et al. 2018). Frequent electrocardiographic changes encompass arrhythmias, ST‐segment elevation, T‐wave inversion and the appearance of pathological Q waves (Liu et al. 2021). Thus, this defining feature is pivotal in nursing care, requiring nurses skilled in identifying and multidisciplinary intervention for such changes. Timely recognition leads to decreased mortality, and continuous in‐hospital monitoring optimises therapeutic management (Diercks et al. 2009; Xiong 2009).

The widespread occurrence of generalised weakness among most patients can be attributed to muscle fatigue, a multifaceted phenomenon involving cellular metabolism, alterations in calcium pumps and changes in myocyte proteins, among other factors (Friedrich 2015). Notably, in the case of the studied patients, with an average age of 65 years, accelerated muscle mass loss occurs after the age of 60. This contributes to muscle fatigue and subsequently limits daily activities (Burgos Peláez 2006).

Given that patients with angina are required to rest during acute episodes, evaluating body composition and mobility should be a routine part of their assessment. Many of these patients are in critical care units, where identifying generalised muscle weakness, muscle retraction, disuse atrophy and myopathy is common. These factors collectively lead to decreased muscle strength, metabolic deterioration, limitations and disability (Jolley, Bunnell, and Hough 2016; Gómez 2012; Schober and Thornton 2013).

Regarding the emotional impact, cardiovascular diseases can influence the perception about the concept of disease. To evaluate our third most prevalent characteristic, discomfort due to exertion, the Broadbent & Cols Short Illness Perception Questionnaire was employed. This instrument provides insight into a patient's perception of their health and its impact (Stein 2014). This tool has found wide application across various health conditions (Broadbent 2006). A study conducted among patients with coronary heart disease by nurses revealed a moderately threatening perception of the disease (Nur 2020). This perception might stem from the symptomatology of coronary heart disease, such as the sensation of impending death. Importantly, negative perceptions of disease impact can directly affect physical and mental health (Vinaccia, Quiceno, and Remor 2012). Nursing support through targeted education programmes can foster more appropriate perceptions and coping strategies for ACS, aiding patient adaptation to their new health condition.

These findings carry significance for nursing professionals in intensive and intermediate care units. The identified characteristics serve as crucial inputs for care plan development and daily patient monitoring. For instance, they inform risk of fall assessments and the application of Barthel dependency scales. Furthermore, patient and family education during visits and discharge regarding activity intolerance and its management strategies is imperative (Pinilla Gomez, Cáceres Manrique, and Orozco Vargas 2010).

This study represents the first clinical validation of the activity intolerance diagnosis in individuals with ACS, enriching the NANDA taxonomy and enhancing nursing practice. The results facilitate the measurement of Nursing Outcomes and the implementation of nursing interventions, including patient and family education, thereby promoting the use of standardised language. The study's strengths encompass the validation of defining characteristics in an under‐studied population, a well‐calculated sample size and an objective, standardised methodology for measuring the defining characteristics. However, certain limitations are worth acknowledging; the research was conducted at a single center. Nevertheless, given FCV's leading status in the region and its reputation as a reference center for this pathology, selection bias is unlikely.

## 5 Conclusions

This study established the clinical validation of the defining characteristics of the nursing diagnosis ‘activity intolerance [00092]’ in patients with ACS, hospitalised in a fourth level care centre specialised in cardiovascular health. As a result, three major defining characteristics were identified: changes in EKG, generalised weakness and discomfort on exertion. These pivotal characteristics should serve as guiding factors in formulating nursing care plans during the acute phase for this specific patient population.

## Author Contributions

Diana Isabel Cáceres Rivera, Luz Mileyde Jaimes Rojas, Diana Ivonne Cañon Gómez and Lyda Z. Rojas were involved in conceptualisation, data curation, formal analysis, methodology, project administration, supervision and writing original draft review and editing. Luisa Yaneth Cristancho Zambrano and Jennifer Vanesa Acosta Barón were involved in, data curation, project supervision and writing original draft review and editing.

## Conflicts of Interest

The authors declare no conflicts of interest.

## Data Availability

Data openly available in a public repository that issues datasets with DOIs.

## References

[nop270050-bib-0001] Abraira, V. 2001. “El Índice Kappa.” Semergen‐Medicina de Familia 27, no. 5: 247–249.

[nop270050-bib-0002] Avezum, Á. , J. Braga , Í. Santos , H. P. Guimarães , and J. A. Marin Neto . 2009. “Cardiovascular Disease in South America: Current Status and Opportunities for Prevention.” Heart 95, no. 18: 1475–1482. 19224906 10.1136/hrt.2008.156331

[nop270050-bib-0003] Broadbent, E. 2006. “The Brief Illness Perception Questionnaire.” Journal of Psychosomatic Research 60, no. 6: 631–637. 16731240 10.1016/j.jpsychores.2005.10.020

[nop270050-bib-0004] Burgos Peláez, R. 2006. “Enfoque Terapéutico Global de la Sarcopenia.” Nutrición Hospitalaria 21, no. suppl. 3: 51–60. http://scielo.isciii.es/scielo.php?script=sci_arttext&pid=S0212‐16112006000600008&lng=es. 16768031

[nop270050-bib-0005] Cáceres, D. , L. Rojas , L. Jaimes , and C. Mileydi . 2022. “Clinical Validation of the Defining Characteristics of the Nursing Diagnosis “Activity Intolerance” in Patients With Acute Coronary Syndrome.” Mendeley Data V1. 10.1002/nop2.70050 PMC11619797 39636748

[nop270050-bib-0006] Cardoso, P. C. , L. Gussatschenko Caballero , K. Brasil Ruschel , M. A. Pereira de Moraes , and E. R. Rabelo da Silva . 2019. “Profile of the Nursing Diagnoses in Stable Heart Disease Patients.” Investigación y Educación en Enfermería 37, no. 2: e08. 10.17533/udea.iee.v37n2e08 PMC7871490 31487445

[nop270050-bib-0007] de Souza, V. Z. 2014. “Content Validation of the Operational Definitions of the Nursing Diagnoses of Activity Intolerance, Excess Fluid Volume, and Decreased Cardiac Output in Patients With Heart Failure.” International Journal of Nursing Knowledge 25, no. 2: 85–93. 24298874 10.1111/2047-3095.12017

[nop270050-bib-0008] Dias Emidio, S. C. , L. Pereira Giovanini , P. R. Gomes Lima , J. Leme Gonçalves , and A. R. de Souza Oliveira . 2021. “Risk for Decreased Cardiac Tissue Perfusion and Activity Intolerance: Association Study.” Avances en Enfermería 39, no. 3: 376–384.

[nop270050-bib-0009] Diercks, D. B. , M. C. Kontos , A. Y. Chen , C. V. Pollack Jr. , S. D. Wiviott , and J. S. Rumsfeld . 2009. “Utilization and Impact of Pre‐Hospital Electrocardiograms for Patients With Acute ST‐Segment Elevation Myocardial Infarction: Data from the NCDR (National Cardiovascular Data Registry) ACTION (Acute Coronary Treatment and Intervention Outcomes Network).” Journal of the American College of Cardiology 53: 161–166. 19130984 10.1016/j.jacc.2008.09.030

[nop270050-bib-0010] Elm, E. V. , D. G. Altman , M. Egger , S. J. Pocock , P. C. Gøtzsche , and J. P. Vandenbroucke . 2007. “Strengthening the Reporting of Observational Studies in Epidemiology (STROBE) Statement: Guidelines for Reporting Observational Studies.” BMJ 335, no. 7624: 806–808. 10.1136/bmj.39335.541782.AD. 17947786 PMC2034723

[nop270050-bib-0011] Fehring, R. J. 1987. “Methods to Validate Nursing Diagnoses.” Heart and Lung: The Journal of Critical Care 16, no. 6 Pt 1: 625–629. 3679856

[nop270050-bib-0012] Friedrich, O. 2015. “The Sick and the Weak: Neuropathies/Myopathies in the Critically Ill.” Physiological Reviews 95, no. 3: 1025–1109. 26133937 10.1152/physrev.00028.2014 PMC4491544

[nop270050-bib-0013] Gómez, W. C. 2012. Fisioterapia en la UCI: Teoría, Experiencia y Evidencia. Bogotá: Editorial El Manual Moderno Colombia.

[nop270050-bib-0014] Grant, J. , M. Kinney , and C. Guzzetta . 1990. “A methodology for Validating Nursing Diagnoses.” Advances in Nursing Science 12, no. 3: 65–74. 2107792 10.1097/00012272-199004000-00007

[nop270050-bib-0015] Jolley, S. E. , A. E. Bunnell , and C. L. Hough . 2016. “ICU‐Acquired Weakness.” Chest 150, no. 5: 1129–1140. 27063347 10.1016/j.chest.2016.03.045 PMC5103015

[nop270050-bib-0016] Liu, J. , R. Wang , B. Wen , Z. Liu , and F. Miao . 2021. “Myocardial Infarction Detection and Localization with Electrocardiogram Based on Convolutional Neural Network.” Chinese Journal of Electronics 30, no. 5: 833–842.

[nop270050-bib-0017] Lunney, M. 2008. “The Need for International Nursing Diagnosis Research and A Theoretical Framework.” International Journal of Nursing Terminologies and Classifications 19, no. 1: 28–34. 18331482 10.1111/j.1744-618X.2007.00076.x

[nop270050-bib-0018] Meek, S. , and F. Morris . 2002. “ABC of Clinical Electrocardiography. Introduction. I‐Leads, Rate, Rhythm, and Cardiac Axis.” British Medical Journal 324, no. 7334: 415–418. 11850377 10.1136/bmj.324.7334.415 PMC1122339

[nop270050-bib-0019] Ministerio de Salud y Protección Social . 2022. “Mortalidad en Colombia Periodo 2020–2021. Medición de la Mortalidad por todas las causas y Covid‐19. Dirección de Epidemiología y Demografía.” https://www.minsalud.gov.co/sites/rid/Lists/BibliotecaDigital/RIDE.

[nop270050-bib-0020] Mol, V. J. , and C. A. Baker . 1991. “Activity Intolerance in the Geriatric Stroke Patient.” Rehabilitation Nursing 16, no. 6: 337–343. 1957055 10.1002/j.2048-7940.1991.tb01244.x

[nop270050-bib-0021] Moreu‐Burgos, J. A.‐M. . 2007. “Fisiopatología del Miocardio Isquémico. Importancia de la Frecuencia Cardiaca.” Revista Española de Cardiología Suplementos 7, no. 4: 19D–25D.

[nop270050-bib-0022] NANDA Internacional . n.d. Diagnósticos enfermeros: Definiciones y Clasificación 2012–2014. Madrid: Elseiver.

[nop270050-bib-0024] Nur, K. R. M. 2020. “Illness Perception and Cardiovascular Health Behaviour Among Persons With Ischemic Heart Disease in Indonesia.” International Journal of Nursing Sciences 5, no. 2: 174–180. 10.1016/j.ijnss.2018.04.007 PMC6626249 31406821

[nop270050-bib-0025] Odenas Alesina, E. , P. Jordán , L. Herrador , et al. 2021. “Q Waves in Ischemic Cardiomyopathy.” International Journal of Cardiovascular Imaging 37, no. 1: 143–146. 10.1007/s10554-021-02172-9 33517554

[nop270050-bib-0026] Padilla‐Garcia, C. I. , S. L. Romero‐Guevara , F. A. Camargo‐Figuera , and A. P. Bonilla‐Marciales . 2017. “Nursing Diagnoses to Hospital Discharge in People With Acute Coronary Syndrome.” MedUNAB 20, no. 1: 19–28.

[nop270050-bib-0027] Paiva, G. D. S. , and M. V. de Oliveira Lopes . 2005. “Respuestas Humanas Identificadas en Pacientes Con Infarto Agudo de Miocardio Ingresados en Una Unidad de Terapia Intensiva.” Enfermería en Cardiología 36: 22–27.

[nop270050-bib-0028] Pereira, J. M. , P. V. Flores , L. D. Figueiredo , et al. 2016. “Nursing Diagnoses of Hospitalized Patients With Heart Failure: A Longitudinal Study.” Revista da Escola de Engermagem da USP 50, no. 6: 929–936. 28198957 10.1590/S0080-623420160000700008

[nop270050-bib-0029] Pinilla Gomez, E. , F. D. M. Cáceres Manrique , and L. C. Orozco Vargas . 2010. “Gradiente Dosis Respuesta de las Intervenciones de Enfermería Para el Control del Temor en Gestantes.” Revista Cubana de Enfermeria 26, no. 2: 27–36.

[nop270050-bib-0030] Puntunet, B. M. L. 2008. “Principales Cuidados de Enfermería en la Persona Con Cardiopatía Isquémica.” Revista Mexicana de Enfermería Cardiológica 16, no. 2: 55–61.

[nop270050-bib-0031] Rodrigues, C. G. , M. A. de Moraes , A. O. Coutinho , J. M. Sauer , R. A. Kalil , and E. N. Souza . 2012. “Clinical Comparison of Patients with Refractory Angina With and Without the Nursing Diagnosis of Activity Intolerance.” Journal of Clinical Nursing 21, no. 23–24: 3579–3582. 23145519 10.1111/j.1365-2702.2011.04059.x

[nop270050-bib-0032] Rodrigues, C. G. , M. A. Moraes , J. M. Sauer , R. A. Kalil , and E. N. de Souza . 2011. “Nursing Diagnosis of Activity Intolerance: Clinical Validation in Patients With Refractory Angina.” International Journal of Nursing Terminologies and Classifications 22, no. 3: 117–122. 21777372 10.1111/j.1744-618X.2011.01182.x

[nop270050-bib-0033] Schober, A. E. , and K. C. Thornton . 2013. “Early Mobilization in the Intensive Care Unit.” Current Anesthesiology Reports 3, no. 2: 73–78.

[nop270050-bib-0034] Siontis, K. C. , P. A. Noseworthy , Z. I. Attia , and P. A. Friedman . 2021. “Artificial Intelligence‐Enhanced Electrocardiography in Cardiovascular Disease Management.” Nature Reviews. Cardiology 18: 465–478. 33526938 10.1038/s41569-020-00503-2 PMC7848866

[nop270050-bib-0035] Souza, V. Z. 2015. “Clinical Usefulness of the Definitions for Defining Characteristics of Activity Intolerance, Excess Fluid Volume and Decreased Cardiac Output in Decompensated Heart Failure. A Descriptive Exploratory Study.” Journal of Clinical Nursing 24, no. 17–18: 2478–2487. 25959208 10.1111/jocn.12832

[nop270050-bib-0036] Stein, A. C. 2014. “Actividad Física y Salud Percibida en Pacientes Con Enfermedad Coronaria.” Cuadernos de Psicología del Deporte 14, no. 1: 109–116.

[nop270050-bib-0037] Strong, A. T. , G. Sharma , C. Tu , et al. 2018. “A Population‐Based Study of Early Postoperative Outcomes in Patients With Heart Failure Undergoing Bariatric Surgery.” Obesity Surgery 28: 2281–2288. 29512040 10.1007/s11695-018-3174-3

[nop270050-bib-0038] Vinaccia, S. , J. M. Quiceno , and E. Remor . 2012. “Resiliencia, Percepción de Enfermedad, Creencias y Afrontamiento Espiritual‐Religioso en Relación Con la Calidad de Vida Relacionada Con la Salud en Enfermos Crónicos Colombianos.” Anales de Psicología/Annals of Psychology 28, no. 2: 366–377.

[nop270050-bib-0039] Whitley, G. G. 1997. “Three Phases of Research in Validating Nursing Diagnoses.” Western Journal of Nursing Research 19, no. 3: 379–399. 9170994 10.1177/019394599701900308

[nop270050-bib-0040] World Health Organization . 2022. “Cardiovascular Diseases.” https://www.who.int/health‐topics/cardiovascular‐diseases#tab=tab_1.

[nop270050-bib-0041] Xiong, P. 2009. “Deep Learning for Detecting and Locating Myocardial Infarction by Electrocardiogram: A Literature Review.” Frontiers in Cardiovascular Medicine 9: 860032. 10.3389/fcvm.2022.860032 PMC8990170 35402563

[nop270050-bib-0042] Zampieron, A. , S. Aldo , and M. Corso . 2011. “A Retrospective Study of Nursing Diagnoses, Outcomes, and Interventions For Patients Admitted to a Cardiology Rehabilitation Unit.” International Journal of Nursing Terminologies and Classifications 22, no. 4: 148–156. 22017735 10.1111/j.1744-618X.2011.01184.x

